# Reduced cardiac function and risk of venous thromboembolism in Asian countries

**DOI:** 10.1186/s12959-017-0135-3

**Published:** 2017-04-24

**Authors:** Ruiqi Zhu, Yu Hu, Liang Tang

**Affiliations:** Department of Hematology, Wuhan Union Hospital of Huazhong University of Science and Technology, Wuhan, 430030 China

## Abstract

Patients with reduced cardiac function are thought to have a higher risk of venous thromboembolism (VTE). Additionally, they are vulnerable to complications of pulmonary embolism (PE) as well as right heart failure (HF), which in return is supposed to increase the rate of mortality. Studies focusing on VTE in heart failure patients were rare in Asian countries before the 21st century. Nowadays, more and more data are becoming available in this field in Asia. It is already known that heart failure can increase the risk of VTE, but so far a consensus on this issue has not been reached for many years, not only in Asian countries but all over the world. This condition may be due to the detailed pathological advancement in Virchow’s triad and some other theories. In clinical practice, VTE, especially PE is difficult to diagnose in patients with heart failure because of overlapping symptoms (e.g. cough and chest pain) and the elevation of laboratory markers (e.g. probrain natriuretic peptide (NT-proBNP) and D-dimer in both heart failure and VTE patients). Management of VTE in heart failure patients is also controversial because heart failure patients always have complications, such as renal failure and hepatic failure, which increase the risk of bleeding. In this study, we analyzed data from China, Japan, Korea, Singapore and India mainly to get a better understanding of the research progress in VTE in patients with heart failure. The aim of this review is to discuss the risk, incidence, advancement of diagnosis, management and prevention of VTE in patients with heart failure in Asian countries.

## Background

Venous thromboembolism (VTE) is considered to be a common global health problem. It includes two major clinical manifestations, namely deep vein thrombosis (DVT) and pulmonary embolism (PE), the latter is commonly generated from DVT, but it is associated with higher mortality. The incidence of VTE was considered to be rare in the Asians population in early publications [1, 2]. It is estimated that the incidence is only about 21–29 cases per 100,000 individuals per year in Asians and Pacific Islanders compared to that of African Americans (138–141 cases per 100,000 individuals per year) and Caucasians (80–117 cases per 100,000 per year) [3, 4]. However, along with the increase in the proportion of elderly people in Asia and the development of diagnostic technology, as well as a better understanding of VTE by Asian clinicians, a huge number of VTE patients have been recognized and the incidence of VTE still shows a rising trend in Asians [5].

Reduced heart function with symptoms of congested lungs, fluid and water retention, rapid or irregular heartbeats as a result of congestive heart failure has recently been determined to be an independent risk factor for VTE in Asians in a SMART study [6]. Additionally, according to a meta-analysis, hospitalized patients with heart failure had an RR of 1.49 (1.16–1.92) for VTE in Asians [7].

Management of patients diagnosed with heart failure and VTE is difficult. Once this kind of patients develop symptoms of heart failure (e.g. dyspnea, cardiogenic shock, elevated jugular venous pressure), symptoms of heart failure may overshadow that of VTE and impede its diagnosis [8]. Moreover, PE increases pulmonary vascular resistance and right ventricular afterload through several mechanisms, including physical obstruction and hypoxemia, which in return deteriorate heart function. In addition, heart failure patients often have severe complications and multiple risk factors that amplify the risk of VTE which make the treatment complicated (e.g., heart failure patients with renal or liver dysfunction have a higher risk of bleeding during or after fibrinolysis) [9]. A study shows that PE with heart failure have a higher overall mortality rate compared to those without heart failure (17% vs 10%) [10] and PE has also been considered as an independent predictor of death in patients with heart failure [10, 11].

## Risk factor and incidence

In Asian patients with heart failure, the incidence of VTE is considered to be lower than that of Western patients. However, some studies have shown that the incidences are neck and neck. It is reported that the incidence of VTE in decompensated heart failure patients ranges from 4 to 26% in Western countries [12], the incidence of PE with congestive heart failure at autopsy ranges from 28 to 48% [13, 14]. A research in Japan reported that the incidence of DVT in heart failure patients was 11.2%, and the incidence is highest in patients classified as NYHA IV (NYHA II: 4.4%, NYHA III: 4.8%, NYHA IV: 25.5%, *P* < 0.01) [15]. One study in Thailand suggested that chronic pulmonary diseases and chronic heart failure were the two most common VTE risk factors in Caucasians, but this study revealed that the highest incidence of VTE was in rheumatologic diseases and the incidence of VTE in congestive heart failure was only 0.5% [16]. This result may due to the study design. The study excluded patients in coronary care units because coronary care unit patients are usually given anticoagulants for cardiac indications, thus the number of heart failure patients in this research is relatively low, the group is too small to get accurate information.

Whether symptomatic reduced heart function is a risk factor for VTE has been a controversial topic for many years, RR for venous thrombosis in patients with heart failure varies from high risk (96.–32.4) [17, 18] to mild risk (1.7–2.6) [19, 20], and even no increase in risk (0.7–0.8) [21, 22]. Data from Asian countries is rare. Nevertheless, it has also shown that Asians have similar non-genetic risk factors as Caucasians, which include severe medical diseases such as heart failure. A prospective, international, multicenter, observational study of a cohort of consecutive Asian patients indicates that congestive heart failure is a risk factor for venous thromboembolic events, whose odds ratio is within the range of Western patients [6]. Some recent studies found that reduced cardiac function is an independent risk factor for VTE in Asians as well as in Caucasians [6, 23–25].

VTE risk is also correlated with the severity of heart failure, as defined by the N-terminal prohormone of brain natriuretic peptide (NT-proBNP) study, which is a multicenter one that was also carried out in China. In this study, the NT-proBNP is suggested to be useful to identify high short-term (Day 10) risk and elevated D-dimer may be useful in recognizing high midterm (Day 35) risk [26]. Elevated NT-proBNP concentration is supposed to be an independent predictor of recurrent VTE in a Chinese study [27], but the result is not consistent with data from the Acute Decompensated Heart Failure National Registry, which indicate that the BNP levels in patients aged ≥65 years is not associated with an increased risk of thromboembolism [28].

## Pathophysiology

**Fig. 1:**
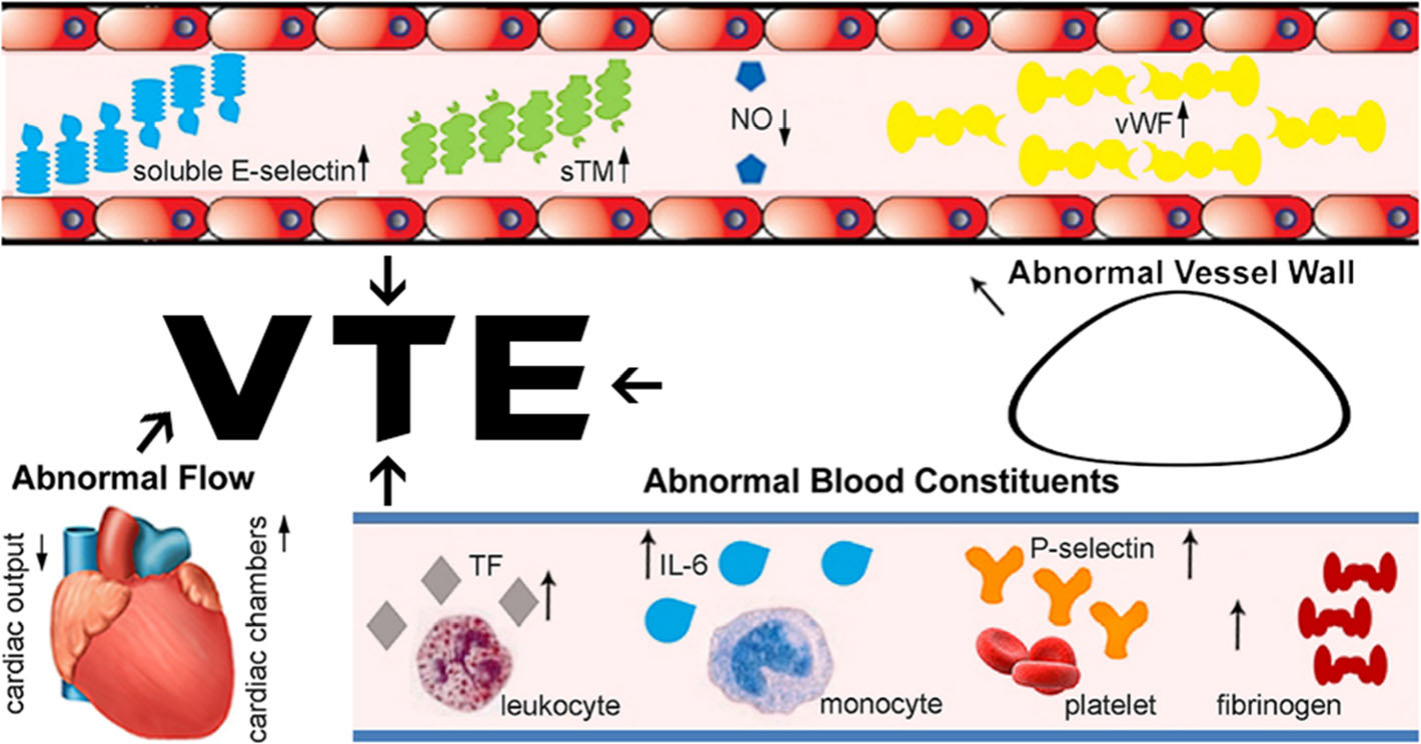
Pathophysiology of thromboembolism in heart failure patients

Virchow’s triad, which originated more than 150 years ago, proposes that endothelial damage and dysfunction, abnormal blood stasis, and a hypercoagulable state are the three main elements for thromboembolism (Fig. 1).

Endothelial damage and dysfunction occurs at the start of endothelial and vascular remodeling, and it is also the start of heart failure in most cases, as most of the heart failure cases are caused by coronary heart disease and hypertension [29], both of them begin with endothelial damage. Endothelial cells stand between the blood vessels and tissues, sense and respond to local hydrodynamics and circulating chemical agents. First, their damage arise as a result of many risk factors for atherosclerosis (e.g. cholesterol, low-density lipoprotein) [30]. Then, endothelial damage is accompanied by pro- and anti-coagulant dysfunction, which puts patients in a pro-thrombotic state. Not only plasma markers of endothelial dysfunction increase in heart failure patients, but also inflammatory cytokines (e.g. interleukin-1) generated by damaged endothelial cell [31].

In addition, heart failure contributes to abnormalities of homeostasis through low cardiac output, the dysfunctional cardiac chambers creates areas of blood stasis, then the abnormalities of homeostasis accelerate the activation of the coagulation system and fibrin formation [31], which makes heart failure patients more vulnerable to VTE.

In addition, increased plasma viscosity, platelet activation, thrombin formation [32] and high levels of circulating coagulant factors and thrombin agents (e.g. fibrinogen, D-dimer) [33] and decreased soluble thrombomodulin [34] in patients with heart failure may lead to a hypercoagulable state and contribute to thrombogenesis.

Although Virchow’s triad explains why heart failure patients are, to some extent, prone to VTE, the exact pathophysiology mechanisms of VTE and heart failure remain to be discovered. Some primary mechanisms include: 1) Impairment of the protein C pathway, specifically, the endothelial protein C receptor (EPCR) is down regulated through inflammatory cytokines elevated in patients with heart failure, which impairs the protein C pathway-mediated anticoagulation [35]. 2) Enhancement of procoagulant reactions resulting in hypercoagulability. 3) Activated receptor (PAR) activation. 4) Neuro-hormonal activation 5) Stasis after low cardiac output [36] and 6) Endothelial dysfunction due to hypoperfusion and systemic inflammation [37]. Tumor necrosis factor and lnterleukin-6 are elevated in heart failure patients [38], the former is associated with the activation of coagulation system and the latter has been shown to be associated with procoagulant tissue factor in heart failure patients [39].

Reduced heart failure patients have increased risk of VTE. In addition, immobilization, infections, central venous catheters and leads from implantable cardiac defibrillators and pacemakers are related to VTE and are common among heart failure patients [9]. Also heart failure and VTE patients both tend to be older [40]. Moreover, in return, acute PE deteriorates the right ventricular (RV) function, which makes the condition complicated and leads to high mortality rates in heart failure patients with VTE.

## Diagnosis of PE in heart failure patients

Reduced cardiac function with PE is likely to go undiagnosed. Indeed, it is often misdiagnosed as worsening heart failure because of the overlapping symptoms and signs and the lack of specific laboratory markers, like dyspnea and D-dimer. Dyspnea occurs both in heart failure and PE patients. The D-dimer laboratory marker is used to help diagnose PE, but it is also elevated in heart failure patients.

Routine imaging examination such as X-ray, electrocardiogram and ultrasonic cardiogram also have specificity problems for the diagnosis of PE. Contrast-enhanced chest computerized tomography (CT) is accurate in the diagnosis of PE.

Diagnosis programs of PE in Asian and Western countries do not show significant difference.

China follows the ESC guidelines on the diagnosis and management of acute PE [41] and the diagnosis program follows three steps: 1) PE possibility assessment 2) Risk stratification 3) Proper examination for diagnosis.

The PE assessment systems used in China are the Wells and Geneva score systems, while the simplified PE severity index (sPESI) is adopted for risk stratification.

However, if patients with heart failure are suspected of suffering from PE after the assessment, they are recommended to undergo contrast-enhanced CT immediately, if they do not have contraindications such as renal failure. The reasons are the followings:

1. In risk stratification strategy, patients who suffer persistent hypotension or shock (systolic pressure under 90 mmHg or drop 40 mmHg) are considered to be high risk and recommended to undergo ultrasonic cardiogram. Ultrasonic cardiogram is used to detect RV overload, however, heart patients can develop RV dysfunction, which makes this examination less useful.
2. For patients who do not suffer persistent hypotension or shock, the D-dimer test or Chest contrast CT is recommended. However, patients with heart failure also have elevated D-dimer levels, which makes it difficult to diagnose PE in heart failure patients.

In brief, a heart failure patient is recommended to take contrast-enhanced chest CT if he/she is highly suspected to have PE.

A previous study in China has shown that in PE patients, heart failure is the third most commonly misdiagnosed disease by clinicians, with a proportion of 8.5%. Additionally, between 2002 and 2006, the number of misdiagnosis of this diseases is four times that between 1984 and 2000, which indicates that the specificity of the various kinds of examinations is not adequate for the diagnosis of PE [42].

The development of molecular biological techniques offers new approaches to help in the diagnosis of this disease. For example, microRNA-134 is reported to be a potential plasma biomarker for the diagnosis of acute PE in Chinese patients [43], but whether it can distinguish PE from heart failure remains to be determined.

In Japan, if a suspected patient has circulatory collapse or cardiopulmonary arrest, or if clinical findings suggest severe risk of PE, the patient is first recommended to receive percutaneous cardiopulmonary support and then undergo contrast CT, pulmonary angiography and transesophageal echocardiography to diagnose PE. If this patient does not have circulatory collapse and clinical findings suggest mild or moderate risk of PE, the D-dimer test is recommended for this patient. If the D-dimer level is high, then the patient should undergo examinations as in the circulatory collapse group [44]. Different conditions of venous thromboembolism in HF patients in Japan and Western Countries are shown in Table 1.

**Table 1:** Different conditions of venous thromboembolism in HF patients in Japan and Western Countries

Items	Japan	Western Countries
Incidence of VTE with HF	11.20%	4%–26%
Risk level of VTE in HF	Moderate	Moderate
Diagnosis		
Steps when suspected PE with shock or hypotension	Percutaneous Cardiopulmonary Support	CT angiography
	Angiography, Echocardiography	Echocardiography
	Treatment or other examine	Treatment or other examine
	43.50%	50.20%
Steps when suspected PE without shock or hypotension	Screening: D-dimer, Echocardiography, X-ray etc.	PE Clinical probability evaluation
	Angiography or MRA or CT angiography	D-dimer
	Treatment or other examine	CT angiography when D-dimer positive
		Treatment or other examine
Prophylaxis rate for prevention	43.50%	50.20%

## Management of PE in China and Japan

Management of PE in reduced cardiac function patients depends on the hemodynamics, right ventricle function and complications in these patients. Conditions are complicated in heart failure patients with severe clinical issues (e.g. renal failure) due to the increased risk of bleeding.

Thus, risk stratifications are also applied in heart failure patients. Various clinical probability scores, calculated according to combinations of known PE risk factors, are used in the management approaches. Heart failure is considered to be the moderate risk level in Asian countries [45, 46], as well as in Western countries.

An agreement has been reached in Asian countries that reduced cardiac function patients who are suffering circulatory collapse should be given hemodynamic and respiratory support immediately. Specifically, percutaneous cardiopulmonary support should be performed according to the Japanese VTE and PE guidelines [44].

Volume loading, limited to 250 to 500 mL, is prescribed in Western countries for systemic arterial hypotensive heart failure patients with PE, without evidence of increased right-sided filling pressures. In China, patients with low cardiac index and normal blood pressure patients are recommended to receive 500 mL of fluid bolus, and this therapy helps to increase cardiac output [9]. However, the Japan VTE guidelines do not suggest volume loading and excessive loading in the right ventricle decreases left cardiac output [44]. Oxygen therapy and drugs, such as dopamine and dobutamine, are also important for the treatment of heart failure patients in all guidelines.

Anticoagulation therapy and thrombolytic therapy are used in PE patients with heart failure without contradictions in China. But in Japan, anticoagulation therapy is for normotensive patients without right heart dysfunction and thrombolytic therapy is the choice for patients with persistent shock and hypotension. In the past, unfractionated heparin was used as anticoagulation agent in Asian countries, just following ESC and ACCP guidelines. However, in 2014, ESC started to strongly recommend non-vitamin K-dependent new oral anticoagulants (NOAC) as an alternative to combined parenteral anticoagulation with a VKA. NOACs have been proven to be not inferior to conditional anticoagulants in efficacy outcome and primary safety outcome and can be used to treat VTE in Western countries. In recent worldwide Hokusai-VTE clinical trial, edoxaban, the oral factor Xa inhibitor, which is a kind of NOACs, administered once daily after initial treatment with heparin was shown noninferior to high-quality standard therapy and could decreased bleeding events significantly [47]. And in patients with right ventricular dysfunction with elevated NT-proBNP levels, a reduction in recurrences was observed in edoxaban group compared with that in warfarin group [47]. When paying attention to Hokusai-VTE trial of East Asia area, Asian patients might get extra benefit from edoxaban compared with warfarin than non-East Asian patients [48]. The major concern was that warfarin may be associated with a higher rate of bleeding in Asians and its efficacy in curing thrombosis was sometimes questioned [49, 50]. Warfarin is difficult to control and is more sensitive in Asians. Edoxaban should be considered an effective and safer alternative to warfarin in East Asian venous thromboembolism patients possessing heart failure at the same time who require anticoagulant treatment. However, data are still limited in Asia, and NOAC treatment is only used in a few medical centers in countries such as China.

Thrombolytic therapy is necessary for reduced heart failure patients because it promptly improves pulmonary circulation [44]. Japan uses monteplase as the only drug officially approved to treat acute PE, whereas in China, the drugs used include alteplase, reteplase and urokinase.

Other treatments for PE do not show significant differences between China and Japan, or between Asian and Western countries. Significant differences in management between areas are shown in Table 2.

**Table 2:** Management shows significant differences between areas

Management	Western countries	China	Japan
Volume loading	250–500 ml	500 ml	None
Anticoagulation therapy	Recommended in PE patients with HF	Recommended in PE patients with HF	For normotensive PE patients without right heart dysfunction
Thrombolytic therapy	Considered for PE patients with HF without contradictions	Recommended in PE patients with HF without contradictions	For PE patients with persistent shock and hypotension
Anticoagulation drugs	NOACs combined parenteral anticoagulation	Unfractionated heparin only except in few medical centers	Unfractionated heparin only except in few medical centers
Thrombolytic drugs	Tenecteplase	Monteplase	Alteplase, reteplase, urokinase

## Prevention

Many VTE guidelines in Asian countries are based on ESC and ACCP guidelines, these guidelines are regarded as ‘gold standard’ in diagnosis, management and prevention of VTE. In 2004, the recommendation grade for the use of anticoagulant prophylaxis in heart failure patients with VTE was Grade 1C+ in the ACCP guidelines, until recently pathophysiological evidence of retrospective data showed that antithrombotic substances can improve outcomes in patients with heart failure. The recommendation grade shifted to Grade 1A. Low-molecular-weight heparin (LMWH), unfractionated heparin (UFH) or fondaparinux (Grade 1A) are recommended in the VTE prophylaxis plan. If anticoagulant prophylaxis is contraindicated, then GCS or IPC should be used.

Although mechanical prophylaxis devices, such as intermittent pneumatic compression devices (IPC), have been shown to be effective in preventing VTE [51, 52], and they are recommended in acutely ill medical patients who have a high bleeding risk in the ACCP guidelines with Grade 1A [53], these devices should be carefully prescribed in cardiac failure patients with VTE, because cardiac function may be worsened by leg compression [5].

Data from some Asian countries also suggest that thromboprophylaxis should be applied in patients with heart failure to prevent VTE events, but the national guidelines for preventing VTE in heart failure patients have not been established in all Asian countries. Recently, consensus has been reached in some Asian countries, including China, Korea, India, etc. [54, 55]. Additionally, VTE prophylaxis rates are high in Asian countries, such as Korea and Japan, but they are still low compared to the higher use of prophylaxis worldwide (50.2%) [56]. The rate in heart/respiratory failure prescribing VTE prophylaxis is estimated to be 46.9% in Korea [55] and 43.5% of heart failure patients received anticoagulant therapy according to a research in Japan [15]. The prophylaxis rates are extremely low in developing countries such as India, although the accurate prophylaxis rate has not been obtained in heart failure patients in this country. In fact, it has been reported that only 19.1% of medical patients received the ACCP recommended thromboprophalaxis [25].

Despite the guidelines recommendation that heart failure patients should receive prophylaxis, a study whose authors including a team in China suggests that only severe heart failure patients whose NT-proBNP concentration is ≥ 1906 pg/ml should receive prophylaxis; less severe heart failure patients do not show a significant difference in VTE incidence compared with patients who do not have heart failure [26]. This study also indicated that rivaroxaban (Clotting Factor X inhibitor) may reduce the risk of VTE in patients with severe heart failure, but enoxaparin (LMWH, 40 mg/d) does not show similar trend [26]. Nevertheless, enoxaparin is widely used in preventing VTE in medically ill patients and several studies provide evidence for the use of enoxaparin. Meanwhile, a study has shown that, in medically ill patients, an extended course of thromboprophylaxis with apixaban is not superior to a shorter course with enoxaparin and the former is significantly associated with more major bleeding events [57]. Another study published in the New England Journal of Medicine in 1999 suggested that enoxaparin is useful to reduce the risk of VTE [58]. Additionally, in the EXCLAIM trial, it was revealed that extended prophylaxis with enoxaparin reduced the rate of VTE in medically ill patients (including congestive heart failure patients) from 4.0 to 0.5%, but increased the rate of major bleeding from 0.3 to 0.8% [59].

In the 2014 Korean guidelines for the prevention of VTE, patients are recommended to be assessed for VTE risk and bleeding risk (Grade 1A, a strong recommendation with high-quality evidence) and prophylaxis is based on risk stratification [60]. Congestive heart failure is considered to be in the moderate risk group for VTE, and may not increase the risk for bleeding according to a study in France [26, 60]. Patients with congestive heart failure only are recommended pharmacological prophylaxis or mechanical prophylaxis, and the recommendation level is Grade 2C (a weak recommendation with low- or very-low-quality evidence). If a patient is admitted to an intensive care unit with multiple risk factors (including heart failure) for VTE, the patient should be routinely assessed and prescribed pharmacological prophylaxis or mechanical prophylaxis (Grade 2A, a weak recommendation with high-quality evidence). Pharmacological prophylaxis mainly comprises LMWH, 0.2–1 mg/kg, subcutaneously daily and low-dose of unfractionated heparin (LDUH), 5000U subcutaneously every 8–12 hours [55]. The recommendation levels in Asian countries are different from the ACCP guidelines, more evidence remain to be discovered in Asian countries.

## Conclusion

In this article, we make a review on the topic of reduced cardiac function and risk of VTE, especially PE in Asian countries in aspects of risk factor, incidence, pathophysiology, diagnosis, management and prevention. Despite genetic risk factors for Eastern and Western countries are different, non-genetic risk factors are similar all over the world. Reduced cardiac function is considered an intermediate risk factor for VTE and should be given attention in Department of Cardiology and Intensive Care Unit. Besides, diagnosis in PE patients with cardiac failure is also intractable because of overlapping of symptoms and signs of these two diseases. Contrast-enhanced chest computerized tomography is necessary when a patient is suspected. Management shows large disparity in different countries. Prevention of PE in Asian countries are mainly based on ESC guideline and developed rapidly in recent years although prophylaxis rate are still lower than western countries. VTE incidence is considered lower than western countries in the past, however emerging evidence suggests the incidence may be nearly the same. Companying with high incidence and mortality in cardiovascular diseases in nowadays Asia, the topic of heart failure and venous thromboembolism should be issued. More studies should be done and a guideline appropriate for Asians is needed.

## Abbreviations

CT: Computerized tomography
DVT: Deep vein thrombosis
ERCP: Endothelial Protein C Receptor
HF: Heart failure
IPC: Intermittent pneumatic compression devices
LDUH: Low-dose of unfractionated heparin
LMWH: Low-molecular-weight heparin
NOAC: Non-vitamin K-dependent new Oral Anticoagulants
NT pro-BNP: Pro-brain Natriuretic Peptide
PAR: Activated receptor
PE: Pulmonary embolism
RV: Right ventricular
sPESI: Simplified PE severity index
UHF: Unfractionated heparin
VTE: Venous thromboembolism
